# Clinical Signs, Staphylococcus and Atopic Eczema-Related Seromarkers

**DOI:** 10.3390/molecules22020291

**Published:** 2017-02-14

**Authors:** Kam Lun Hon, Kathy Yin Ching Tsang, Jeng Sum C. Kung, Ting Fan Leung, Christopher W. K. Lam, Chun Kwok Wong

**Affiliations:** 1 Department of Pediatrics, The Chinese University of Hong Kong, Prince of Wales Hospital, Hong Kong, China; kathytsang78@link.cuhk.edu.hk (K.Y.C.T.); jsckung@gmail.com (J.S.C.K.); tfleung@cuhk.edu.hk (T.F.L.); 2 State Key Laboratory of Quality Research in Chinese Medicine, Macau Institute for Applied Research in Medicine and Health, Macau University of Science and Technology, Taipa, Macau, China; wklam@must.edu.mo; 3 Department of Chemical Pathology, The Chinese University of Hong Kong, Prince of Wales Hospital, Shatin, NT, Hong Kong, China; ck-wong@cuhk.edu.hk; 4 Institute of Chinese Medicine and State Key Laboratory of Phytochemistry and Plant Resources in West China, The Chinese University of Hong Kong, Hong Kong, China

**Keywords:** atopic dermatitis, anti-staphylococcal enterotoxin IgE, eosinophil, IgE, antigen presenting cell, CDLQI, SCORAD, T helper and Treg lymphocytes

## Abstract

Childhood eczema or atopic dermatitis (AD) is a distressing disease associated with pruritus, sleep disturbance, impaired quality of life and *Staphylococcus aureus* isolation. The pathophysiology of AD is complex and various seromarkers of immunity are involved. We investigated if anti-staphylococcal enterotoxin IgE (anti-SE), selected seromarkers of T regulatory (Treg), T helper (Th) and antigen-presenting cells (APC) are associated with clinical signs of disease severity and quality of life. Disease severity was assessed with the Scoring Atopic Dermatitis (SCORAD) index, and quality of life with the Children’s Dermatology Life Quality Index (CDLQI) in AD patients ≤18 years old. Concentrations of anti-*staphylococcus* enterotoxin A and B immunoglobulin E (anti-SEA and anti-SEB), selected Treg/Th/APC chemokines, skin hydration and transepidermal water loss (TEWL) were measured in these patients. Forty patients with AD [median (interquartile range) age of 13.1 (7.9) years) were recruited. Backward stepwise linear regression (controlling for age, personal allergic rhinitis and asthma, and other blood markers) showed the serum anti-SEB level was positively associated with *S. aureus* and *S. epidermidis* isolations, objective SCORAD, clinical signs and CDLQI. TNF-α (a Th1 cytokine) was positively associated with objective SCORAD (B = 4.935, *p* = 0.010), TGF-β (a Treg cytokine) negatively with disease extent (B = −0.015, *p* = 0.001), IL-18 (an APC cytokine) positively with disease extent (B = 0.438, *p* = 0.001) and with TEWL (B = 0.040, *p* = 0.010), and IL-23 (an APC cytokine) negatively with disease extent (B = −2.812, *p* = 0.006) and positively with pruritus (B = 0.387, *p* = 0.007). Conclusions: Blood levels of anti-SEB, Th1, Treg and APC cytokines are correlated with various clinical signs of AD. AD is a systemic immunologic disease involving *Staphylococcus aureus*, cellular, humoral, cytokine and chemokine pathophysiology.

## 1. Introduction

Eczema or atopic dermatitis (AD) is a distressing disease associated with pruritus, sleep disturbance and impaired quality of life [1,2]. AD is a common and often persistent skin disease that affects a large percentage of the world’s population [1]. Atopy refers to allergic hypersensitivity that is associated with asthma, inhalant allergies (e.g., hay fever), and chronic dermatitis. There is a known hereditary component of the disease, and it is more common in atopic families [1]. Criteria that enable a doctor to diagnose it include the typical appearance and distribution of the rash in a patient with a personal or family history of atopy [1,3,4].

AD is a challenge for modern medicine because it is prevalent, severely affects the quality of life of patients and their families, and causes high socioeconomic costs [5]. While initial studies suggested a Th2 deviation as the primary reason for the disease, numerous studies addressed a genetically predetermined impaired epidermal barrier as the leading cause in a subgroup of patients. Recently, immune changes beyond the initial Th2 concept were defined in AD, with a role for specialized dendritic cells as well as newly identified T helper lymphocyte subsets such as Th17 and Th22 lymphocytes. Furthermore, trigger factors have been expanded beyond classical Th2 allergens such as pollen or house dust mites to microbial products as well as self-antigens.

AD pathophysiology is complex and involves the interplay of various T helper (Th) and T regulatory (Treg) lymphocytes and chemokines [1,6,7,8,9]. Significant advances in our understanding of the pathogenesis of AD have led to improvements in therapy. Assessment of AD and the therapeutic efficacy of various treatments is based on objective clinical scores and measurements [10]. These assessments include measurement of skin parameters (such as skin hydration, transepidermal water loss, erythema, staphylococcal colonization and isolation) and laboratory measurement of AD-related seromarkers [10,11].

Many Th2 chemokines and cytokines are involved in the inflammatory process and have been intensely studied [8]. Correlations with disease severity are demonstrated in C-C motif chemokines (such as CCL17, CCL18, CCL22), interleukins (such as IL-17 and IL-31) and neuropeptides (such as brain-derived neurotrophic factor and substance P) [6,7,12,13]. *Staphylococcus aureus* is a key pathogen in the complex pathophysiology of AD [14,15,16]. *Staphylococcal* enterotoxin B (SEB) has been demonstrated to mediate the disease process [17]. Quantifying the various markers and correlating their levels with clinical scores should lead to a better understanding of AD and improve research and efficacy in its management. We investigated correlations among serum levels of anti-staphylococcal enterotoxin IgE, selected AD-related seromarkers and various clinical parameters of AD.

## 2. Results

From December 2012 to August 2014, 40 children with AD (23 boys and 17 girls) were recruited (Table 1). The median (IQR) age of patients was 13.1 (7.9) years. Their median (IQR) objective Scoring Atopic Dermatitis (SCORAD) and Children Dermatology Life Quality Index (CDLQI) scores, and mean ± SD skin hydration (SH) and transepidermal water loss (TEWL) were 45.0 (11.7), 10.0 (8.0), 32.8 ± 13.5 and 12.1 ± 1.7, respectively. There were 16 (40%) and 24 (60%) patients with moderate and severe AD, respectively [18]. Thirty-six (90%) and seventeen (43%) patients were found to have skin colonized with *S. aureus* or *S. epidermidis*, and sixteen of these patients were colonized with both bacteria. A significantly higher percentage of eosinophils in the differential white cell count (*p* = 0.039), the logarithm of serum total IgE (*p* = 0.008), and the serum concentrations of IFN-γ (*p* = 0.005) and IL-18 (*p* = 0.007) were observed in patients with severe AD compared to those with moderate AD (Table 2).

**Figure 1:**
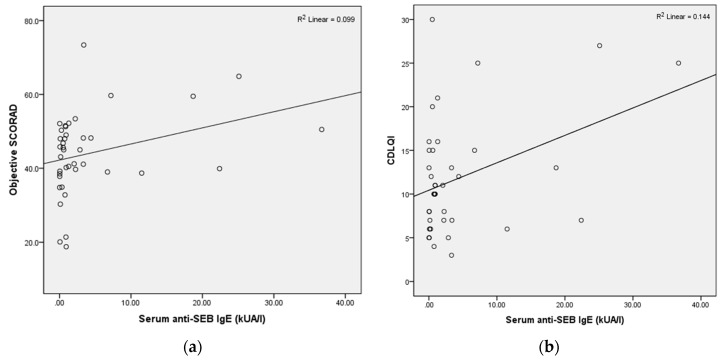
Scatterplots of serum anti-SEB IgE with (**a**) objective SCORAD and (**b**) CDLQI.

Spearman’s correlations (adjusted for age of admission, personal allergic rhinitis and asthma) between blood markers and clinical indices are summarized in Table 3. Scatterplots showed weak associations between the serum anti-SEB IgE level with both objective SCORAD and CDLQI (Figure 1), and these associations still existed after regression with adjustment.

Backward stepwise linear regression (controlling for age of admission, personal allergic rhinitis and asthma, and other blood markers) showed the serum anti-SEB IgE level was positively associated with *S. aureus* (B = 0.027, *p* = 0.046) and *S. epidermidis* (B = 0.033, *p* = 0.043) isolations, objective SCORAD (B = 0.445, *p* = 0.007), clinical signs (disease extent (B = 0.554, *p* = 0.006) and intensity (B = 0.099, *p* = 0.012)) and CDLQI (B = 0.421, *p* < 0.0005). Serum TNF-α (a Th1 cytokine) was associated with objective SCORAD (B = 4.935, *p* = 0.010) (Table 4). Associations were also demonstrated with disease extent and IL-18 (an APC cytokine; B = 0.438, *p* = 0.001), TGF-β (a Treg cytokine; B = −0.015, *p* = 0.001) and IL-23 (B = −2.812, *p* = 0.006); pruritus and IL-23 (an APC cytokine, B = 0.387, *p* = 0.007); TEWL and IL-18 (B = 0.040, *p* = 0.010).

## 3. Discussion

The pathogenesis of atopic dermatitis involves complex interactions between susceptible genes, immunological status, skin barrier defects, infections, and environmental factors [1,8]. Activation of T lymphocytes, dendritic cells, macrophages, keratocytes, mast cells, and eosinophils is characteristic of AD skin inflammatory responses. In particular, it involves the interplay of APC, Th2 and Th1 lymphocytes and chemokines [1,8]. Some of these cells and seromarkers have been demonstrated to correlate closely with clinical signs and scores [6,7,19].

AD is a clinical diagnosis with characteristic itchy skin lesions in a child with xerosis, and a personal or family history of atopy. Clinical investigations are usually not necessary for its diagnosis. The disease is diagnosed according to criteria proposed by Hanifin and Rajka [20], or by the UK working diagnostic criteria [21]. Various domains or parameters of the disease, such as symptomatology, signs, severity and quality of life can be objectively measured [22,23,24]. The disease is readily treated by avoidance of allergens and application of topical emollients, corticostoerids or immunomodulating agents [1,2]. However, there is no immediate cure for the disease. Hence, it is pivotal to manage expectations of the parents and patients.

### 3.1. Anti-*Staphylococcus* Enterotoxins Immunoglobulin E (anti-SE IgE)

Bacterial infection, most commonly with *S. aureus*, is the main complication of AD [1,25]. In fact, *Staphylococcus* is pathophysiologically associated with AD [14,15]. The anti-SE IgE responses to *Staphylococcus* have not been well characterized. We demonstrated that the serum anti-SEB IgE level and not anti-SEA IgE was positively associated with *S. aureus* and/or *S. epidermidis* isolations, objective SCORAD, clinical signs and CDLQI. In vivo, staphylococcal enterotoxin B but not viruses or Th1 and Th2 cytokines induced IL-31 in leukocytes [26]. Our findings therefore provide a new link among staphylococcal isolation, subsequent T cell recruitment/activation, and symptom induction in patients with atopic dermatitis.

### 3.2. Th Cytokines

AD involves defective cell-mediated immunity related, in part, to an imbalance in two subsets of CD4- T cells that creates a predominance of T-memory cells in the Th2 pathways and a preferential apoptosis of IFN-γ producing Th1 memory and effector T cells [27]. Th2 cells express a set of cytokines, IL-4, -5, -6, -10 and -13 [28]. These cytokines stimulate the proliferation and differentiation of B lymphocytes and contribute to the hypereosinophilia, high serum IgE level, sustained cutaneous inflammation, histamine release, and pruritus characteristic of the disorder [29]. Compared with extrinsic atopic dermatitis, the intrinsic form is associated with less IL-4 and IL-13 production. Maintenance of chronic inflammation is associated with the predominance of IL-5 and IL-12 expression and eosinophils [29].

T helper lymphocytes can be sub-classified on the basis of their repertoire of cytokines [6,30]. The Th1 family produces IFN-γ, TNF-β, TNF-α, IL-2, Th2 produces IL-4, IL-5, IL-9, IL-13, IL-25, GM-CSF, IL-3, IL-10, and Treg produces TGF-β, IL-10. Th1 cells activate T lymphocytes and monocytes and promote cell-mediated immune responses; Th2 cells produce IL-4, IL-5 and IL-13 and function in the relative absence of IFN-γ to induce allergic immune responses. Among the many Th1 cytokines that have been evaluated, we demonstrated in this study that TNF-α was independently and positively associated with objective SCORAD.

Chemokines are leukocyte chemoattractive proteins of 8–10 kDa that represent the largest family of cytokines encoded in the human genome [6,31]. They are critically important in regulating leukocyte trafficking in vivo and participate in the activation of leukocytes in tissues. The CC and CXC chemokines are involved in mediating allergic inflammation. Although not specifically included in the multiplex kit in the current study, these chemokines have been demonstrated to correlate closely with disease severity [6]. These molecules interact closely in a bi-directional manner with Th1 and Th2 cytokines under the adaptive immune system. For example, CC chemokines regulate the directed locomotion of Th2 cells and eosinophils to sites of allergic inflammation and their secretion is also affected by Th2 cytokines such as IL-4 and IL-13. Chemokines also work closely with the innate immune system, and the activation of which, as mediated through the pattern recognition receptors CD14 and TLRs, results in the increased expression of chemokines and their receptors on a variety of leukocytes.

### 3.3. Treg Cytokines

Regulatory T lymphocytes (Treg) produce the immunosuppressive cytokines transforming growth factor (TGF)-β and IL-10 that are important in actively suppressing or terminating immune responses [32,33,34]. We demonstrated that serum TGF-β of AD children inversely correlated with disease extent. IL-10 appeared to correlate with objective SCORAD, and the extent and intensity of skin lesions, but the associations became insignificant in the linear regression analysis. This phenomenon is complicated and may be explained by *S. aureus* isolation (as colonization/infection). *S. aureus* promotes inflammation in the skin of AD children through concomitant Th2 activation and the silencing of resident Treg cells [35]. Commensals such as *S. epidermidis* may counteract these effects by inducing the release of IL-10 by skin dendritic cells [15,35]. The continuous presence of *Staphylococcus* enterotoxin B (SEB) can trigger an acquired functional impairment of Treg in AD patients and the correlation between the increased frequency of Treg and disease severity supports their important role in AD pathogenesis [17].

### 3.4. APC Cytokines

The production of chemokines by antigen-presenting cells (such as CCL18) is enhanced by Th2 cytokines such as IL-4 and IL-13 and suppressed by IFN-γ [12,36,37,38]. Two APC cytokines were investigated in this study. IL-18 is a pleiotrophic APC cytokine that plays an important role in both Th1 and Th2 helper T lymphocyte–mediated immunity [39]. The present study found that the plasma concentration of IL-18 positively correlated with the extent or area of the disease, whereas IL-23, also an APC cytokine, negatively correlated with the extent but positively with pruritus. IL-18, also designated as an IFN-γ–inducing factor, was originally considered a Th1 cytokine by acting through its ability to induce IFN-γ production [40]. Recent studies, however, indicated a more complicated pleiotrophic role for IL-18 than simply the induction of IFN-γ production. IL-18 was reported to induce the production of IgE and Th2 cytokines, and is associated with the pathogenesis and severity of AD in children and adults [39,41,42,43,44,45].

### 3.5. Quality of Life

Quality of life, as evaluated with CDLQI, is an important independent domain in the study of atopic disease [24]. CDLQI has been reported to correlate with several clinical and laboratory parameters [10,14,24]. In this study we confirmed that the atopic factors, namely personal history of asthma, eosinophilia and serum concentration of anti-SEB IgE, were positively and independently associated with CDLQI. These findings provided a research direction towards management of these atopic factors for improving the quality of life of patients with eczema.

In conclusion, we have demonstrated that serum concentrations of anti-SEB IgE as well as Th1, Treg and APC cytokines were associated with various clinical signs and quality of life in AD. Together with previous reports on Th2, other immune cellular players and *Staphylococcus aureus*, we confirmed AD is truly a systemic immunologic disease involving *Staphylococcus*, cellular (e.g., APC, eosinophils, neutrophils, basophils, Th1, Th2, Treg, B cells), humoral, cytokine and chemokine pathophysiology [6,19,46]. As the inflammatory process in AD is complex, it may be impossible to characterize the disease with a single clinical score or seromarker. Quantifying the various markers and correlating their levels with clinical scores may lead to a better understanding of AD and improve research in its management. The availability of relevant laboratory markers is an important additional armamentarium for the ongoing research and treatment of this miserable disease in children. The effects of AD treatment on clinical and immune markers can be studied.

## 4. Patients and Methods

AD patients of Chinese ethnicity <18 years of age were recruited from the pediatric dermatology clinic of a university teaching hospital (Hong Kong, China). AD was diagnosed according to standard criteria [3,20] and its severity and symptomatology including pruritus and sleep loss were evaluated using SCORAD index [18,21]. The SCORAD has been widely employed in AD research. It combines objective signs of extent and intensity and subjective symptoms of pruritus and sleep loss. The objective SCORAD, on the other hand, eliminates the subjective symptoms and provides a severity grading [18]. There is no standard global parameter for assessment of eczema severity but SCORAD is one of the most frequent scores usual in clinical medicine. Quality of life of these AD patients was measured with a validated Chinese version of the Children Dermatology Life Quality Index (CDLQI) [24,47,48]. CDLQI consists of 10 questions and has been extensively translated into many languages and validated. Skin biophysiologic parameters (skin hydration (SH) and transepidermal water loss (TEWL)) were measured by standardized procedure at the right antecubital fossa using the Corneometer CM 825 and the Tewameter TM 210 (Courage Khazaka Electronic GmbH, Cologne, Germany), respectively [49]. Also, skin swabs (Copan Innovation, Brescia, Italy) were collected from the right antecubital fossa and the worst affected site (i.e., lesional skin with oozing or crusting) of the patients by rubbing each area for 5 s [14,15].

Peripheral venous blood was collected from AD patients. Part of the fresh blood was collected into EDTA and used for measuring peripheral eosinophil percentage using the Coulter STKS counter (Beckman-Coulter, Miami, FL, USA). The remaining blood was allowed to clot and centrifuged at 4 °C and 3000× *g* for 10 min and the resulting serum stored immediately at −80 °C until further analysis. Serum total IgE was quantified using micro-particle immunoassay (IMx analyzer, Abbott Laboratories, Chicago, IL, USA). All experiments were conducted according to the manufacturer’s specifications.

Anti-SEA IgE and anti-SEB IgE were measured by ImmunoCAP fluorescent enzyme immunoassays (Cat. No. 14-4889-01 (m80) and 14-4890-01 (m81)), which was conducted by loading all the required chemicals in a sequential order to the automatic system Phadia 100 (Phadia, Uppsala, Sweden) according to the manufacturer’s instructions. After loading the chemicals, a sequence of reactions would proceed automatically by the system. SEA (m80) (or SEB (m81)) covalently bound to the solid phase would first react with the anti-SEA IgE (or anti-SEB IgE) in the serum sample. After washing away the unbound IgE, β-galactosidase-conjugated mouse monoclonal anti-IgE antibodies were added to form complex with the specific IgE. Following the removal of unbound anti-IgE antibodies, 0.01% 4-methylumbelliferyl α-d-galactopyranoside was added for fluorescent signal development with the help of β-galactosidase. The enzymatic reaction was then stopped by adding 4% sodium carbonate. The fluorescent signal emitted at the end-point was measured by the system, and the serum level of the specific IgE was calculated automatically based on the standard curve. A serum specific IgE level of 0.35 kU/L or greater was considered positive to the specific allergen sensitization.

Concentrations of other seromarkers IFN-γ, TNF-α, IL-9, IL-10, IL-12, IL-18 and IL-23 were measured using the ProcartaPlex magnetic bead-based multiplex assay (Cat. No. EPX180-12165-901; eBioscience, Vienna, Austria); while TGF-β concentration was determined with the ProcartaPlex magnetic bead-based simplex assay (Cat. No. EPX010-10249-901). 50 μL of magnetic beads coated with cytokine-specific capture antibodies was added to each well of a ProcartaPlex 96-well flat bottom plate, followed by 2 min incubation to coat the plate with the magnetic beads, and aspiration by washing buffer to remove unbound beads. Then, 25 μL of serum samples or standards and 25 μL of universal assay buffer were added to each well. The plate was incubated with shaking for 2 h, which allowed the cytokines to bind to their respective immobilized specific magnetic beads. After aspiration, 25 μL of biotinylated cytokine-specific detection antibody was added, followed by incubation with shaking for 30 min to allow binding to the bead-cytokine complex. Following aspiration was the addition of 50 μL of Streptavidin-Phycoerythrin (SA-PE) and incubation with shaking for 30 min to allow the binding between biotin and SA. All incubations were carried out at room temperature in the dark. Finally, the plate was aspirated again, and 120 μL of reading buffer was added to re-suspend the beads from the bottom of each well. The plate containing the bead complex suspensions was read using Bio-Plex 200 (BioRad; Hercules, CA, USA). The concentrations of different cytokines in each serum sample could then be calculated automatically based on the respective standard curves. The simplex assay was conducted in a similar way as the multiplex assay, except that serum samples needed to be pre-treated with 1 N HCl and 1.2 N NaOH/0.5 M HEPES to convert the inactive form of TGF-β to the active form of TGF-β1 because only the bioactive form of TGF-β was immunoreactive and detectable.

Data were expressed as proportion, or mean and standard deviation (SD) (or median and interquartile range (IQR) if data were not normally distributed). Pearson’s chi-squared test (or Fisher’s exact test if more than 20% of cells with expected frequencies less than 5) and independent samples *t*-test (or Mann-Whitney U test if data were not normally distributed) were used to analyze categorical and numerical variables between groups, respectively. The correlations between different blood markers and clinical indices were analyzed by Spearman’s correlation coefficients. Backward stepwise linear regression was conducted using IBM SPSS 20.0 to investigate the independent associations. All comparisons were made two-tailed, and *p*-values less than 0.05 were considered to be statistically significant. Informed written consent was obtained from parents, and Clinical Research Ethics Committee of our University approved this study.

## Figures and Tables

**Table 1:** Demographics and clinical parameters of patients with moderate and severe AD.

		Total (*n* = 40)	Moderate AD (*n* = 16)	Severe AD (*n* = 24)	*p*
Demographics					
Age, years		13.1 (7.9)	12.8 (7.2)	13.7 (6.5)	0.345 *
Sex–Male, *n* (%)		23 (57.5)	10 (62.5)	13 (54.2)	0.601
Personal allergic rhinitis, *n* (%)		28 (70.0)	10 (62.5)	18 (75.0)	0.490 **
Personal asthma, *n* (%)		19 (47.5)	6 (37.5)	13 (54.2)	0.301
Clinical parameters					
Objective SCORAD		45.0 (11.7)	38.2 (8.7)	48.6 (6.5)	<0.0005 *
*Extent*, %		54.7 ± 14.8	43.9 ± 13.0	61.8 ± 11.2	<0.0005
*Intensity*		9.5 (3.0)	8.0 (2.0)	10.5 (2.0)	<0.0005 *
Pruritus		6.7 ± 1.9	6.6 ± 1.9	6.8 ± 1.9	0.786
Sleep loss		5.9 ± 2.8	5.9 ± 2.6	5.9 ± 3.0	0.946
CDLQI		10.0 (8.0)	10.0 (8.0)	10.5 (9.0)	0.594 *
SH		32.8 ± 13.5	37.4 ± 13.2	29.7 ± 13.0	0.078
TEWL, g/m^2^/h		12.1 ± 1.7	11.7 ± 1.3	12.4 ± 1.8	0.181
*S. aureus*, *n* (%) ^#^	No	4 (10.0)	2 (12.5)	2 (8.3)	0.184 **
	Scanty	18 (45.0)	10 (62.5)	8 (33.3)	
	Moderate	15 (37.5)	3 (18.8)	12 (50.0)	
	Heavy	3 (7.5)	1 (6.2)	2 (8.3)	
*S. epidermidis*, *n* (%) ^#^	No	23 (57.5)	8 (50.0)	15 (62.5)	0.150 **
	Scanty	11 (27.5)	7 (43.8)	4 (16.7)	
	Moderate	6 (15.0)	1 (6.2)	5 (20.8)	

AD, atopic dermatitis; CDLQI, Children’s Dermatology Life Quality Index; SCORAD, SCORing Atopic Dermatitis; SH, skin hydration; TEWL, transepidermal water loss. Numerical data expressed in either mean ± standard deviation or median (interquartile range) depending on the distribution normality. * Mann-Whitney *U* test; ** Fisher’s exact test; ^#^ Bacterial isolation in the worst affected skin area.

**Table 2:** Concentrations of seromarkers in patients with moderate and severe AD.

Blood/Serum Markers	Total (*n* = 40)	Moderate AD (*n* = 16)	Severe AD (*n* = 24)	*p*
Eosinophil % in WBC	10.0 (7.0)	9.0 (6.0)	10.5 (6.0)	0.039 *
log (Total IgE count in kU/L)	3.79 ± 0.51	3.53 ± 0.57	3.96 ± 0.39	0.008
Anti-SEA IgE, kU/L	0.81 (2.68)	0.37 (2.64)	1.17 (2.66)	0.279 *
Anti-SEB IgE, kU/L	0.90 (3.15)	0.83 (1.96)	1.10 (2.87)	0.202 *
IFN-γ, pg/mL	5.83 (6.24)	4.76 (2.86)	9.04 (6.79)	0.005 *
TGF-β, pg/mL	607 (530)	804 (526)	558 (489)	0.192 *
TNF-α, pg/mL	1.90 (1.07)	1.74 (1.21)	2.06 (0.91)	0.165 *
IL-9, pg/mL	0.71 (1.16) (*n* = 34)	0.41 (0.89) (*n* = 12)	0.71 (1.11) (*n* = 22)	0.276 *
IL-10, pg/mL	0.51 (0.58) (*n* = 29)	0.41 (0.53) (*n* = 9)	0.63 (0.45) (*n* = 20)	0.077 *
IL-12, pg/mL	0.44 (0.20)	0.37 (0.10)	0.44 (0.2)	0.079 *
IL-18, pg/mL	18.15 (15.95)	13.68 (9.50)	22.96 (20.81)	0.007 *
IL-23, pg/mL	1.08 (0.46)	1.08 (0.46)	1.08 (0.46)	0.946 *

AD, atopic dermatitis; IFN, interferon; IL, interleukin; SE, staphylococcus enterotoxin; TGF, transforming growth factor; TNF, tumor necrosis factor; WBC, white blood cell. Data expressed in either mean ± standard deviation or median (interquartile range) depending on the distribution normality. * Mann-Whitney *U* test.

**Table 3:** Adjusted Spearman’s correlations between seromarkers and clinical indices (*n* = 40; adjusted for age of admission, personal allergic rhinitis and personal asthma).

		*S. aureus* Density *	*S.* *epidermidis* Density *	Objective SCORAD	Extent, %	Intensity	Pruritus	Sleep Loss	CDLQI	SH	TEWL, g/m^2^/h
*S. aureus* isolation *	rho		0.093	**0.436**	0.237	**0.459**	**0.503**	0.208	0.007	−0.198	0.280
	*p*		0.583	**0.007**	0.158	**0.004**	**0.002**	0.217	0.969	0.240	0.094
*S.* *epidermidis* isolation *	rho			0.045	0.103	0.013	**0.342**	0.209	0.134	0.044	−0.012
	*p*			0.791	0.545	0.938	**0.038**	0.215	0.428	0.797	0.942
Eosinophil % in WBC	rho	**0.478**	0.015	**0.476**	**0.362**	**0.448**	0.295	0.275	**0.425**	**−0.370**	−0.024
	*p*	**0.003**	0.928	**0.003**	**0.028**	**0.005**	0.077	0.100	**0.009**	**0.024**	0.889
log(Total IgE count in kU/L)	rho	**0.436**	0.053	**0.552**	**0.423**	**0.518**	**0.415**	0.179	**0.405**	**−0.350**	0.122
	*p*	**0.007**	0.756	**0.000**	**0.009**	**0.001**	**0.011**	0.288	**0.013**	**0.034**	0.473
Anti-SEA IgE, kU/L	rho	0.051	−0.083	0.214	−0.009	0.209	0.173	−0.097	**0.399**	−0.032	−0.107
	*p*	0.764	0.626	0.204	0.957	0.214	0.306	0.566	**0.014**	0.850	0.528
Anti-SEB IgE, kU/L	rho	0.167	0.031	**0.361**	0.290	**0.342**	0.206	0.031	**0.360**	**−0.416**	0.105
	*p*	0.324	0.854	**0.028**	0.081	**0.038**	0.221	0.856	**0.029**	**0.010**	0.538
IFN-γ, pg/mL	rho	**0.339**	−0.120	**0.531**	**0.530**	**0.516**	0.324	0.204	−0.047	−0.227	0.193
	*p*	**0.040**	0.478	**0.001**	**0.001**	**0.001**	0.050	0.225	0.780	0.177	0.252
TGF-β, pg/mL	rho	−0.110	0.108	−0.191	−0.259	−0.122	−0.007	0.098	0.157	0.084	−0.091
	*p*	0.517	0.523	0.258	0.121	0.473	0.968	0.564	0.353	0.619	0.592
TNF-α, pg/mL	rho	0.231	−0.038	**0.422**	**0.593**	**0.371**	0.258	0.264	0.070	−0.132	0.066
	*p*	0.168	0.825	**0.009**	**0.000**	**0.024**	0.123	0.114	0.680	0.437	0.697
IL-9, pg/mL (*n* = 34)	rho	−0.065	−0.201	0.202	**0.562**	0.115	0.123	0.282	−0.138	−0.184	0.072
	*p*	0.730	0.279	0.275	**0.001**	0.536	0.509	0.124	0.460	0.322	0.702
IL-10, pg/mL (*n* = 29)	rho	0.228	−0.084	**0.554**	**0.571**	**0.468**	−0.043	0.218	0.007	−0.002	−0.100
	*p*	0.264	0.684	**0.003**	**0.002**	**0.016**	0.836	0.284	0.974	0.994	0.627
IL-12, pg/mL	rho	0.254	−0.071	**0.390**	**0.570**	**0.331**	0.234	0.060	−0.204	−0.196	0.215
	*p*	0.129	0.676	**0.017**	**0.000**	**0.045**	0.164	0.723	0.226	0.244	0.201
IL-18, pg/mL	rho	**0.426**	0.042	**0.587**	**0.547**	**0.562**	**0.380**	0.261	0.022	−0.121	0.268
	*p*	**0.009**	0.804	**0.000**	**0.000**	**0.000**	**0.020**	0.119	0.899	0.474	0.109
IL-23, pg/mL	rho	0.309	0.016	0.202	0.301	0.166	**0.389**	**0.338**	−0.080	−0.083	0.042
	*p*	0.063	0.925	0.231	0.070	0.327	**0.017**	**0.041**	0.637	0.623	0.807
SH	rho	−0.198	0.044	**−0.352**	**−0.356**	**−0.337**	−0.200	−0.267	−0.320		0.163
	*p*	0.240	0.797	**0.033**	**0.031**	**0.041**	0.234	0.111	0.054		0.335
TEWL, g/m^2^/h	rho	0.280	−0.012	0.214	0.158	0.215	0.277	0.205	−0.225		
	*p*	0.094	0.942	0.203	0.349	0.200	0.097	0.223	0.181		

Significant correlations are highlighted in bold. * Bacterial isolation in the worst affected skin area: no growth, scanty, moderate and heavy growth.

**Table 4:** Results of backward stepwise linear regression (controlling for age of admission, personal allergic rhinitis and asthma, and other blood markers that had rho ≤−0.2 or ≥0.2 with the clinical index).

***S. aureus*** **Isolation**	% of eosinophils in blood white cell counts (B = 0.083, *p* = 0.001), serum concentration of anti-SEB IgE (B = 0.027, *p* = 0.046) and TEWL (B = 0.224, *p* = 0.004) were positively and independently associated with *S. aureus* isolation.
***S. epidermidis*** **Isolation**	Serum concentration of anti-SEB IgE (B = 0.033, *p* = 0.043) was positively associated with *S. epidermidis* isolation.
**Objective SCORAD**	% of eosinophils in blood white cell counts (B = 0.843, *p* = 0.003), serum concentrations of anti-SEB IgE (B = 0.445, *p* = 0.007) and TNF-α (B = 4.935, *p* = 0.010) were positively and independently associated with objective SCORAD.
**Extent**	Serum concentrations of anti-SEB IgE (B = 0.554, *p* = 0.006) and IL-18 (B = 0.438, *p* = 0.001) were positively and independently associated with extent, while serum concentrations of TGF-β (B = −0.015, *p* = 0.001) and IL-23 (B = −2.812, *p* = 0.006), and SH (B = −0.605, *p* = 0.001) were negatively and independently associated with extent.
**Intensity**	Presence of personal asthma (B = 1.265, *p* = 0.044), % of eosinophils in blood white cell counts (B = 0.197, *p* = 0.004), serum concentration of anti-SEB IgE (B = 0.099, *p* = 0.012) and TEWL (B = 0.421, *p* = 0.040) were positively and independently associated with intensity.
**Pruritus**	*S. aureus* density (B = 0.815, *p* = 0.004) and *S. epidermidis* density (B = 0.709, *p* = 0.010), and serum concentration of IL-23 (B = 0.387, *p* = 0.007) were positively and independently associated with pruritus, while age of admission (B = −0.216, *p* < 0.0005) was negatively associated with pruritus.
**Sleep Loss**	*S. epidermidis* density (B = 1.478, *p* = 0.038) was positively associated with sleep loss, while age of admission (B = −0.392, *p* = 0.002) and presence of personal asthma (B = −2.386, *p* = 0.027) were negatively and independently associated with sleep loss.None of the studied blood markers were independently associated with sleep loss.
**CDLQI**	Presence of personal asthma (B = 3.441, *p* = 0.041), % of eosinophils in blood white cell counts (B = 0.482, *p* = 0.004) and serum concentration of anti-SEB IgE (B = 0.421, *p* < 0.0005) were positively and independently associated with CDLQI; while age of admission (B = −0.361, *p* = 0.050) and presence of personal allergic rhinitis (B = −9.269, *p* < 0.0005) were negatively and independently associated with CDLQI.
**SH**	None of the studied blood markers were independently associated with SH.
**TEWL**	Serum concentration of IL-18 (B = 0.040, *p* = 0.010) was positively associated with TEWL.

## References

[1] Leung A.K., Hon K.L., Robson W.L. (2007). Atopic dermatitis. Adv. Pediatr..

[2] Leung T.N., Hon K.L. (2015). Eczema therapeutics in children: what do the clinical trials say?. Hong Kong Med. J..

[3] Williams H.C., Burney P.G., Pembroke A.C., Hay R.J. (1994). The U.K. Working Party’s Diagnostic Criteria for Atopic Dermatitis. III. Independent hospital validation. Br. J. Dermatol..

[4] Williams H.C., Burney P.G., Hay R.J., Archer C.B., Shipley M.J., Hunter J.J., Bingham E.A., Finlay A.Y., Pembroke A.C., Graham-Brown R.A. (1994). The U.K. Working Party’s Diagnostic Criteria for Atopic Dermatitis. I. Derivation of a minimum set of discriminators for atopic dermatitis. Br. J. Dermatol..

[5] Eyerich K., Novak N. (2013). Immunology of atopic eczema: Overcoming the Th1/Th2 paradigm. Allergy.

[6] Hon K., Leung T.F. (2010). Seromarkers in childhood atopic dermatitis. Expert Rev. Dermatol..

[7] Hon K.L., Ching G.K., Wong K.Y., Leung T.F., Leung A.K. (2008). A pilot study to explore the usefulness of antibody array in childhood atopic dermatitis. J. Natl. Med. Assoc..

[8] Homey B., Steinhoff M., Ruzicka T., Leung D.Y. (2006). Cytokines and chemokines orchestrate atopic skin inflammation. J. Allergy Clin. Immunol..

[9] Hayashida S., Uchi H., Moroi Y., Furue M. (2011). Decrease in circulating Th17 cells correlates with increased levels of CCL17, IgE and eosinophils in atopic dermatitis. J. Dermatol. Sci..

[10] Hon K.L., Kung J.S., Wang M., Pong N.H., Li A.M., Leung T.F. (2016). Clinical scores of sleep loss/itch and antihistamine/topical corticosteroid usage for childhood eczema. Br. J. Dermatol..

[11] Hon K.L., Luk C.K., Tsang Y.C., Pong N.H., Leung T.F. (2016). Objective measurement of two clinical signs in childhood atopic eczema in research and therapeutics. J. Dermatolog. Treat..

[12] Hon K.L., Ching G.K., Ng P.C., Leung T.F. (2011). Exploring CCL18, eczema severity and atopy. Pediatr. Allergy Immunol..

[13] Hon K.L., Lam M.C., Wong K.Y., Leung T.F., Ng P.C. (2007). Pathophysiology of nocturnal scratching in childhood atopic dermatitis: The role of brain-derived neurotrophic factor and substance P. Br. J. Dermatol..

[14] Hon K.L., Tsang Y.C., Pong N.H., Ng C., Ip M., Leung T.F. (2016). Clinical features and Staphylococcus aureus colonization/infection in childhood atopic dermatitis. J. Dermatol. Treat..

[15] Hon K.L., Tsang Y.C., Pong N.H., Leung T.F., Ip M. (2016). Exploring Staphylococcus epidermidis in atopic eczema: Friend or foe?. Clin. Exp. Dermatol..

[16] Hon K.L., Tsang Y.C., Lee V.W., Pong N.H., Ha G., Lee S.T., Chow C.M., Leung T.F. (2016). Efficacy of sodium hypochlorite (bleach) baths to reduce Staphylococcus aureus colonization in childhood onset moderate-to-severe eczema: A randomized, placebo-controlled cross-over trial. J. Dermatol. Treat..

[17] Gaspar K., Barath S., Nagy G., Mocsai G., Gyimesi E., Szodoray P., Irinyi B., Zeher M., Remenyik É., Szegedi A. (2015). Regulatory T-cell subsets with acquired functional impairment: Important indicators of disease severity in atopic dermatitis. Acta Derm. Venereol..

[18] Kunz B., Oranje A.P., Labreze L., Stalder J.F., Ring J., Taieb A. (1997). Clinical validation and guidelines for the SCORAD index: Consensus report of the European Task Force on Atopic Dermatitis. Dermatology.

[19] Hon K.L., Wang S.S., Pong N.H., Leung T.F. (2013). Circulating Immunoglobulins, Leucocytes and Complements in Childhood-onset Atopic Eczema. Indian J. Pediatr..

[20] Hanifin J., Rajka G. (1980). Diagnostic features of atopic dermatitis. Acta Derm. Venereol. (Stockh.).

[21] (1993). Severity scoring of atopic dermatitis: The SCORAD Index. Consensus Report of the European Task Force on Atopic Dermatitis. Dermatology.

[22] Charman C., Williams H. (2000). Outcome measures of disease severity in atopic eczema. Arch. Dermatol..

[23] Charman C., Chambers C., Williams H. (2003). Measuring atopic dermatitis severity in randomized controlled clinical trials: What exactly are we measuring?. J. Investig. Dermatol..

[24] Hon K., Kam W.Y., Lam M., Leung T., Ng P.C. (2006). CDLQI, SCORAD and NESS: Are they Correlated?. Qual. Life Res..

[25] Hon K.L., Lam M.C., Leung T.F., Kam W.Y., Li M.C., Ip M., Fok T.F. (2005). Clinical features associated with nasal Staphylococcus aureus colonisation in Chinese children with moderate-to-severe atopic dermatitis. Ann. Acad. Med. Singap..

[26] Sonkoly E., Muller A., Lauerma A.I., Pivarcsi A., Soto H., Kemeny L., Alenius H., Dieu-Nosjean M.C., Meller S., Rieker J. (2006). IL-31: A new link between T cells and pruritus in atopic skin inflammation. J. Allergy Clin. Immunol..

[27] Leung A.K.C., Barber K.A. (2003). Managing childhood atopic dermatitis. Adv. Ther..

[28] Leung D.Y., Jain N., Leo H.L. (2003). New concepts in the pathogenesis of atopic dermatitis. Curr. Opin. Immunol..

[29] Abramovits W. (2005). Atopic dermatitis. J. Am. Acad. Dermatol..

[30] Mosmann T.R., Coffman R.L. (1989). TH1 and TH2 cells: Different patterns of lymphokine secretion lead to different functional properties. Ann. Rev. Immunol..

[31] Luster A.D. (1998). Chemokines—Chemotactic cytokines that mediate inflammation. N. Eng. J. Med..

[32] Sakaguchi S. (2000). Regulatory T cells: Key controllers of immunologic self-tolerance. Cell.

[33] Lee N., Shin J.U., Jin S., Yun K.N., Kim J.Y., Park C.O., Kim S.H., Noh J.Y., Lee K.H. (2016). Upregulation of CD47 in Regulatory T Cells in Atopic Dermatitis. Yonsei Med. J..

[34] Roesner L.M., Floess S., Witte T., Olek S., Huehn J., Werfel T. (2015). Foxp3(+) regulatory T cells are expanded in severe atopic dermatitis patients. Allergy.

[35] Laborel-Preneron E., Bianchi P., Boralevi F., Lehours P., Fraysse F., Morice-Picard F., Sugai M., Sato’o Y., Badiou C., Lina G. (2015). Effects of the *Staphylococcus aureus* and *Staphylococcus epidermidis* Secretomes Isolated from the Skin Microbiota of Atopic Children on CD4+ T Cell Activation. PLoS ONE.

[36] Kodelja V., Muller C., Politz O., Hakij N., Orfanos C.E., Goerdt S. (1998). Alternative macrophage activation-associated CC-chemokine-1, a novel structural homologue of macrophage inflammatory protein-1 alpha with a Th2-associated expression pattern. J. Immunol..

[37] Vulcano M., Struyf S., Scapini P., Cassatella M., Bernasconi S., Bonecchi R., Calleri A., Penna G., Adorini L., Luini W. (2003). Unique regulation of CCL18 production by maturing dendritic cells. J. Immunol..

[38] De Nadai P. (2004). Involvement of CCL18 in Inflammatory Allergic Diseases. J. Allergy Clin. Immunol..

[39] Hon K.L.E., Leung T.F., Ma K.C., Wong C.K.P., Wan H.M.R.C., Lam C.W. (2004). Serum Concentration of IL-18 Correlates with Disease Extent in Young Children with Atopic Dermatitis. Pediatr. Dermatol..

[40] Okamura H., Tsutsui H., Komatsu T., Yutsudo M., Hakura A., Tanimoto T., Torigoe K., Okura T., Nukada Y., Hattori K. (1995). Cloning of a new cytokine that induces IFN-gamma production by T cells. Nature.

[41] Yoshizawa Y., Nomaguchi H., Izaki S., Kitamura K. (2002). Serum cytokine levels in atopic dermatitis. Clin. Exp. Dermatol..

[42] El Mezzein R.E.H., Matsumoto T., Nomiyama H., Miike T. (2001). Increased secretion of IL-18 in vitro by peripheral blood mononuclear cells of patients with bronchial asthma and atopic dermatitis. Clin. Exp. Immunol..

[43] Higashi N.M., Gesser B.P., Kawana S.M., Thestrup-Pedersen K.M.P. (2001). Expression of IL-18 mRNA and secretion of IL-18 are reduced in monocytes from patients with atopic dermatitis. J. Allergy Clin. Immunol..

[44] Hoshino T., Wiltrout R.H., Young H.A. (1999). IL-18 is a potent coinducer of IL-13 in NK and T cells: A new potential role for IL-18 in modulating the immune response. J. Immunol..

[45] Wild J.S., Sigounas A., Sur N., Siddiqui M.S., Alam R., Kurimoto M., Sur S. (2000). IFN-gamma-inducing factor (IL-18) increases allergic sensitization, serum IgE, Th2 cytokines, and airway eosinophilia in a mouse model of allergic asthma. J. Immunol..

[46] Wong C.K., Chu I.M., Hon K.L., Tsang M.S., Lam C.W. (2016). Aberrant Expression of Bacterial Pattern Recognition Receptor NOD2 of Basophils and Microbicidal Peptides in Atopic Dermatitis. Molecules.

[47] Chuh A.A. (2003). Validation of a Cantonese version of the Children’s Dermatology Life Quality Index. Pediatr. Dermatol..

[48] Lewis-Jones M.S., Finlay A.Y. (1995). The Children’s Dermatology Life Quality Index (CDLQI): Initial validation and practical use. Br. J. Dermatol..

[49] Hon K.L., Wong K.Y., Leung T.F., Chow C.M., Ng P.C. (2008). Comparison of Skin Hydration Evaluation Sites and Correlations among Skin Hydration, Transepidermal Water Loss, SCORAD Index, Nottingham Eczema Severity Score, and Quality of Life in Patients with Atopic Dermatitis. Am. J. Clin. Dermatol..

